# Notes and News

**Published:** 1892-11-05

**Authors:** 


### NOTES AND NEWS.

COLLECTED AND COMMUNICATED.

The Duke of Devonshire, who laid the foundation-stone of the older portion of the Chesterfield and North Derbyshire Hospital in 1859, then accommodating but twelve patients, has just shown his continued interest in the institution by presenting £500 towards the cost of a new wing. The usefulness and popularity of the hospital are increasing very greatly, and the proposed addition is undoubtedly needed.

What a sanitary age we live in! Last week the system of ventilation at the Queen's Bench Division of the Law Courts went wrong; and Mr. Justice Day, then sitting, discovered on inquiry that, owing to hermetically sealed windows, nothing but stale air was being inhaled by those present. Rather than subject the Court to such an atmosphere he adjourned for the day. Did the learned judge, we wonder, read our warnings against accumulated carbonic acid gas in a recent issue of The Hospital?

The *Oban Times* of the 24th ult. gives an interesting sketch of the home life of the poor in the West Highlands. The picture of the state of their dwellings when occupied by the whole family and miniature farm population is very startling to those who imagine something of a more healthy, if not a less rude manner of living amongst the highlanders. The article states that provision for ventilation or drainage is rare. Cattle and fowls are sometimes kept inside the dwelling, and the manure and other refuse is frequently kept stored for some time before removal. What this portends may be imagined, when we consider that the floors consist of mud, often below the level of the outside ground. If boards are occasionally raised above this they simply serve to cover a foul cesspool. Those who have visited the Highlands will know that the picture is not an exaggerated one, and were it not for the large amount of time spent out of doors the Highlanders would be far from the hardy race they are. No doubt that consumption would not flourish amongst them as it does under healthier home conditions.

The Committee appointed at the meeting at Spencer House to consider the question of a Central Hospital Board held its first meeting on Wednesday, and elected Lord Sandhurst chairman.

We learn that Mr. Henman's excellent plans for the new General Hospital, Birmingham, are undergoing a slight alteration, as a very complete scheme of mechanical ventilation is to be carried out in the building. This new hospital should be worthy of the city which takes so just a pride in its charitable institutions, as no pains are being spared to make it so. The building will be only partially commenced this year, as all the site is not yet available.

A most important addition has just been made to the Chester Infirmary. The new wing, named after Colonel Humbertson, was opened last week by the Duke of Westminster. It is to be devoted entirely to nurses' accommodation, and to domestic offices. The sum expended on the building, amounting to £2,500, was contributed by the late Colonel Humbertson, and by public subscription. Colonel Humbertson had been for twenty-five years chairman of the Board of Management, and had also been Mayor of Chester.

The site of the new wing at St. Mary's Hospital is preparing apace. The demolition of the wretched little habitations which previously occupied the ground reveals to us the hospital much more clearly than the crowded neighbourhood has made possible before, and convinces the observer that the new departure will be a beneficial one, if only by bringing the institution within sight of the crowds who traverse the narrow thoroughfare of Praed Street. We do not know within what distance of the present pavements the new buildings are to advance, but we hope that the designers have arranged a sufficiently large free space in front, both for the purpose of showing the erection to greater advantage and to improve the thoroughfare. We learn that the foundation-stone is to be laid on December 17th, on which occasion a presentation of purses containing each not less than five guineas is to be made in aid of the new Clarence Memorial Wing.

We have on previous occasions drawn attention to the benefit the possession of a piano confers upon patients in hospitals by assisting to brighten many a weary hour. In several cases where we have appealed on behalf of some one institution kind friends have come forward and supplied the want. It is within the power of some of the large musical instrument manufacturers to assist in this good object, and we do not doubt they would respond, were they effectively appealed to. Such gifts are especially suitable to the approaching Christmastide, when hearts are especially ready to give pleasure where they can. This year we ask assistance on behalf of the Royal Albert Edward Infirmary, Wigan, situated in that weird black country, which may be interesting at night with its lurid glare from countless fires to the passing traveller esconced in a comfortable railway carriage, and on his way to more congenial regions, but which affords a terrible surrounding to the poor. To them a sojourn in the hospital can be made a pleasant memory to look back upon by the help of a little brightness within its walls, and those anxious to attain this object would be greatly assisted were a piano forthcoming.

